# Hyponatremia-induced psychosis in an industrial setting

**DOI:** 10.4103/0972-6748.62277

**Published:** 2009

**Authors:** Rakesh Kumar Singh, Suprakash Chaudhury

**Affiliations:** Department of Psychiatry, Ranchi Institute of Neuropsychiatry and Allied Sciences, Kanke, Ranchi, Jharkhand, India

The Editor,

Symptoms of hyponatremia can range from increased psychotic symptoms to seizures, coma and even death. To observe the incidence of complications in severely hyponatremic hospitalized patients and relate outcome to rate of correction, all patients admitted to a tertiary referral hospital in New York City and a group of hospitals in Oxford with a sodium ≤ 120 mmol/L were studied. There were 84 episodes in New York and 100 in Oxford over a period of 9.5 months and 1 year, respectively; 79% had chronic hyponatremia (>3 days’ duration). During hyponatremia, 76% of patients had clouding of consciousness, with 11% in coma. Other hyponatremic complications included long track signs (including hemiparesis) (6.0%), seizures (3.3%), hallucinations (0.5%), tremor (1.0%), intellectual impairment without clouding of consciousness (0.5%) and acute psychosis (0.5%). As many as 4.3% of the patients died as a direct result of their electrolyte disturbance. After correction, central pontine myelinolysis (0.5%), post-correction seizures (1.0%), intellectual impairment (2.2%), tremor (0.5%), paresthesiae (0.5%) and striatal syndrome (0.5%) were observed. Correction of hyponatremia was started in 158 patients, and the mean maximum rate of correction in 24 hours was 8.4 mmol/L (SD, 5.6; range, 2-42). The maximum rate of correction was higher in those who developed neurological sequelae (12.1 mmol/L/24 h *vs*. 8.2 mmol/L/24 h; *P* = .0125, *t* test, separate variance, two-tail). Neurological sequelae were associated with faster rates of correction, and correction of chronic severe hyponatremia should be < 10 mmol/L in 24 hours (Ellis, 1995). Over a 3-year period, 15 patients with severe hyponatremia were referred to our emergency room from a nearby psychiatric institution. This article reports on 36 episodes of symptomatic hyponatremia in those 15 patients. All but two of the patients were receiving antipsychotic medications; 1 patient was taking a nonsteroidal anti-inflammatory drug, and 1 patient was taking an oral hypoglycemic agent. Thirteen patients were chronic schizophrenics, 1 patient had a bipolar depressive disorder with psychotic features and 1 patient had no psychiatric disorder. Patients presented with seizures, change in mental status, and vegetative symptoms (nausea, vomiting and diarrhea) associated with hyponatremia and water intoxication. Exacerbation of the patients’ underlying illness, psychogenic polydipsia, compulsive smoking, alcoholic cirrhosis, drug abuse and neuroleptic and other medications are thought to be the major causes of acute hyponatremia in these patients (Ellinas *et al*., 1993). Hyponatremia has also been reported as an unusual side effect of drugs like tricyclic antidepressants (Sharma and Pompei, 1996), thiazides (Haensch *et al*., 1996), carbamazepine (Schmitz and Trimble, 1995) and lisinopril (Giupponi and Erfurth, 1998). Here we report a case of an industrial worker without a past or family history of psychiatric disorder who developed psychotic symptoms due to hyponatremia caused probably by dehydration.

An employee, 34-year-old, Hindu, married male, working in a large aluminum plant had symptoms characterized by intense perspiration, weakness, lethargy precipitated by higher temperature in the plant. Within few minutes, he was seen to be behaving abnormally in the form of irrelevant talk, poor reality testing and perplexity. He was brought to the emergency department of the factory hospital. Vitals were normal. Investigation in the form of hemoglobin, total leukocyte count, differential leukocyte count, blood for malarial parasite, and serum electrolytes was ordered. Results of all investigations were normal except serum sodium of 111 mEq/L. He was given IV sodium chloride 2 pint over 3 hours. With this, there was marked improvement in the abnormal behavior. He was completely normal after 6 hours and discharged and counseled for increased salt intake due to heat and the nature of his job. This case history depicts hyponatremia-induced psychosis.

Fluid-electrolyte balance is regulated within a narrow range, and disturbances in this system are unusual in animals and humans. Studies from the preneuroleptic era to date suggest that up to 25% of patients with schizophrenia have polydipsia, suggesting that it is related to the pathophysiology of the psychoses. Polydipsia and the related phenomenon of hyponatremia cause considerable mortality and morbidity. Prevalence studies are limited by imprecise measures available at present. The treatment was limiting water intake when patients reached critical levels of water retention, which however did not improve polydipsia. Recent case reports and open studies have shown that clozapine improves both polydipsia and water retention. The response occurs at low doses and is not related to improvement in psychosis. This may not be applicable to all patients, and better understanding of the pathophysiology of polydipsia-hyponatremia would lead to more empirically derived treatments (Verghese *et al*., 1996). The unique feature of the present case is that unlike most cases reported in the literature, the patient was not suffering from any psychiatric disorder and had no past or family history of psychiatric disorders. He developed hyponatremia due to dehydration secondary to high temperatures in the workplace. However, the response to appropriate therapy was prompt and complete.
